# Segmentation study of nanoparticle topological structures based on synthetic data

**DOI:** 10.1371/journal.pone.0311228

**Published:** 2024-10-02

**Authors:** Fengfeng Liang, Yu Zhang, Chuntian Zhou, Heng Zhang, Guangjie Liu, Jinlong Zhu

**Affiliations:** School of Computer Science and Technology, Changchun Normal University, Changchun, China; University of Salento, ITALY

## Abstract

Nanoparticles exhibit broad applications in materials mechanics, medicine, energy and other fields. The ordered arrangement of nanoparticles is very important to fully understand their properties and functionalities. However, in materials science, the acquisition of training images requires a large number of professionals and the labor cost is extremely high, so there are usually very few training samples in the field of materials. In this study, a segmentation method of nanoparticle topological structure based on synthetic data (SD) is proposed, which aims to solve the issue of small data in the field of materials. Our findings reveal that the combination of SD generated by rendering software with merely 15% Authentic Data (AD) shows better performance in training deep learning model. The trained U-Net model shows that Miou of 0.8476, accuracy of 0.9970, Kappa of 0.8207, and Dice of 0.9103, respectively. Compared with data enhancement alone, our approach yields a 1% improvement in the Miou metric. These results show that our proposed strategy can achieve better prediction performance without increasing the cost of data acquisition.

## Introduction

Nanomaterials exhibit distinctive characteristics and possess a broad spectrum of applications, showcasing their impact even at the minutest scales within realms such as cosmetics, textiles, and food industries. Furthermore, their significance extends across a diverse array of technologies, encompassing domains like medicine, electronics, and energy, where they assume pivotal roles [1–5]. Precise comprehension and manipulation of nanomaterial structures are imperative for harnessing their unique attributes effectively. The attributes of nanoparticles, including their dimensions, morphology, and surface chemistry, not only influence product quality [6, 7] but also hold paramount importance in assessing their interactions with molecules, cells, and broader biological systems. These attributes are integral for conducting comprehensive evaluations of environmental and human health risks associated with nanomaterials [8]. Serving as the fundamental constituents of nanomaterials, nanoparticles demand thorough structural analysis to unravel their properties and functionalities effectively.

Nanoparticles serve as the fundamental constituents of nanomaterials, and scrutinizing their structure is imperative for elucidating the properties and functionalities of such materials. Electron Microscopy (EM) [9] stands as the predominant technique for characterizing particle structure, including Transmission Electron Microscopy (TEM) [10], Scanning Electron Microscopy (SEM) [11], and Atomic Force Microscopy (AFM) [12]. Various methods [13–19] have been proposed for automating image analysis by SEM and TEM. Batuhan Yildirim et al. [20] introduced an automated Bayesian deep learning approach for electron microscope image analysis, facilitating the extraction of quantitative metrics such as particle size. Khuram Faraz et al. [21] devised a deep learning-based method coupled with computer vision for tracking nanoparticles in ambient transmission electron microscopy (ETEM) sequences, enabling objective and robust analysis of dynamic events, particularly relevant to heterogeneous catalytic reactions. Paul Monchot et al. [22] addressed particle size characterization in scanning electron microscope (SEM) images through the use of the Mask-RCNN algorithm in deep learning, surpassing limitations of conventional image processing methods and offering a high-performance solution for automated processing chains. Zhijian Sun et al. [23] achieved precise nanoparticle segmentation through a lightweight deep learning network (NSNet), facilitating rapid and accurate statistical analysis of nanoparticle morphology in complex SEM/TEM images. D.J. Groom et al. [24] explored the implementation of an automatic particle pickup device based on the variance-mixed-mean local thresholding method, effectively enhancing nanoparticle segmentation accuracy in transmission electron microscope (TEM) images by reducing false detections and omissions. Bastian Rühle et al. [25] demonstrated automatic segmentation of agglomerated non-spherical nanoparticles in scanning electron microscope images, eliminating the necessity for large-scale manually labeled training datasets.

In recent years, owing to the continuous evolution of deep learning and machine learning methodologies, there has emerged the capability to precisely extract feature information from images for nanoparticle analysis utilizing these sophisticated techniques [11, 26, 27]. Nevertheless, as the majority of these methodologies rely on supervised learning paradigms, a substantial amount of human effort is necessitated for data preparation, which is crucial for model training. The primary challenge lies in acquiring a representative dataset of nanoparticle images. While approaches such as “exact learning” [28], “transfer learning” [29], and data augmentation techniques mitigate the need for extensive training data, they still entail significant human intervention in data curation. Moreover, these methods often entail errors, consume considerable time, and incur high costs. To alleviate this predicament, a recent trend involves the utilization of SEM and TEM images as training data for deep learning-driven nanoparticle analysis. For instance, Binbin Lin et al. [30] utilized the Mask R-CNN algorithm, particularly for segmentation, employing Geodict software to synthesize a considerable quantity of nanowires. Meanwhile, Leonid Mill et al. [31] leveraged rendering software to create lifelike synthetic training data, crucial for training cutting-edge deep neural networks. This innovation enables automated and high-throughput particle detection across various imaging techniques. Simultaneously, Antón Cid-Mejías et al. [32] accomplished the successful detection, segmentation, orientation inference, and three-dimensional reconstruction of nanoparticles within microscope images by employing artificially synthesized image datasets resembling authentic nanoparticle photographs. This methodology presents a groundbreaking approach for swift and precise nanoparticle characterization. In another study, Lehan Yao et al. [33] Seamlessly integrated liquid-phase transmission electron microscopy with a U-Net neural network-based analysis framework, thereby automating the efficient analysis of nanoparticle behavior in liquid-phase TEM videos by simulating training data from TEM images. This approach divulges pivotal insights into the dynamics of synthetic and biological nanomaterials at the nanoscale. Additionally, Simon Müller et al. [34] employed a 3D U-Net architecture to reliably segment volumetric images of electrodes, remedying the deficiencies of traditional methods in scenarios with inadequate contrast. The network’s performance was enhanced by synthesizing learning data, successfully applied to segment X-ray chromatography microscopy images of graphite-silicon composite electrodes, enabling statistical analysis of microstructural evolution during battery operation. Moreover, Leonid Mill et al. [35] introduced SYNTA as an innovative approach to providing training data for deep learning systems by generating synthetic, lifelike biomedical images. Demonstrating versatility in muscle fiber and histological section analysis, they showcased robust segmentation tasks achievable on previously unseen AD using solely synthetic training data, potentially expediting biomedical image analysis. Furthermore, Boyuan Ma et al. [36] proposed a transfer learning strategy addressing the challenge of limited or simulated data by integrating real and simulated data and expanding training through data mining. In a grain image segmentation task, a model trained with only 35% AD alongside acquired SD achieved segmentation performance comparable to a model trained with all AD.

In summary, current nanoparticle research predominantly emphasizes the segmentation analysis of nanoparticles themselves, with less attention directed towards the segmentation study of nanoparticle structure. While SD offers a swift and efficient means of exploring nanoparticle structure, existing synthetic datasets often rely on expensive professional material modeling software to accurately replicate real TEM images. In our study, we eschew costly professional material modeling software and instead utilize the most basic 3D modeling software to generate synthetic images. The experimental findings indicate that a mere 15% of AD suffices for improved nanoparticle segmentation outcomes, achieving a Dice coefficient of up to 0.91. This experiment underscores the feasibility of reasonably evaluating the performance of a model or experimental system based on real-life scenes by leveraging a small amount of AD. Concurrently, employing abundant SD enables the extension of dataset scale and diversity, covering a broader array of scenarios and feature combinations, thereby enhancing the generalizability of research findings. This integrated approach, leveraging both real and SD, presents an effective and cost-efficient method for investigating nanoparticle structures, facilitating the attainment of desired experimental outcomes and the advancement of scientific research.

## Materials and methods

Our objective is to derive diverse insights into nanoparticle structures from high-resolution microscopic images acquired via electron or ion microscopy, necessitating a representative quantity of training data. However, prevailing hardware constraints pose a significant challenge as accessible microscope image data typically falls short in supporting robust training of deep convolutional neural networks (CNNs). A solution advocated in this study involves amalgamating a limited amount of AD with a substantial volume of SD to attain the requisite sample size and diversity for experimental purposes. This strategy not only mitigates research expenses but also safeguards the credibility and robustness of experiments.

### Dataset establishment

**Fig 1:**
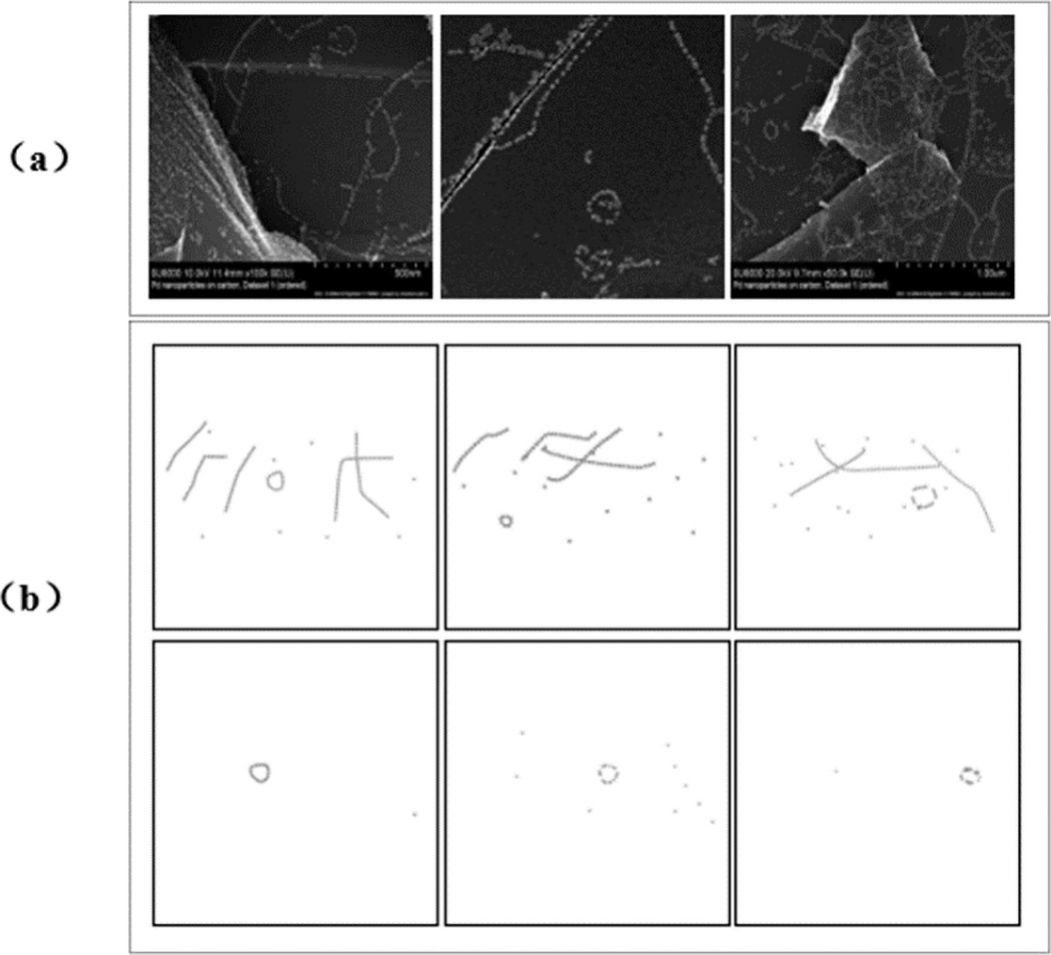
Two dataset presentations. (a) is AD; (b) is SD.

The genuine dataset utilized in this investigation originates from BOIKO’s [37] ordered dataset 1, acquired via electron microscopy and comprising 750 images. These images depict nanoparticles adhering to a carbon surface in an organized fashion, revealing various geometrical patterns. Observations include the presence of curved and straight line formations, individual nanoparticles detached from surrounding structures, contrasting gradations from light to dark, and the presence of large luminous particles, often forming extensive arrays or contaminants. These characteristics are exemplified by the AD depicted in Fig 1. In our study, our primary focus was on segmenting nanoparticle structures with circular formations. To streamline the analysis process and minimize ambiguity, we deliberately selected images exhibiting clearly defined circular rings, as the inclusion of mixed image types would complicate the analysis. This selection criterion is illustrated in Fig 1(a).

For the SD, we employed K-3D [38], the fundamental 3D model design software, as a rendering tool to generate images portraying nanoparticle structures closely resembling their real counterparts. As illustrated in the two synthesized data forms depicted in Fig 1(b), the first image meticulously mimics AD to provide an authentic reflection of the real-world scene, whereas the second image is synthesized with the objective of optimizing nanoparticle circle segmentation by simplifying image components, thereby yielding more precise results.

In this study, the real and synthetic datasets undergo initial labeling utilizing the labelme tool, followed by extensive data augmentation procedures. These enhancements encompass diverse transformations, including random horizontal flipping, vertical adjustments, mirror symmetry manipulations, affine alterations, rotations, Gaussian noise addition, contrast modifications, scale transformations, panning, and more. Subsequently, from the augmented datasets, 400 samples are designated for training purposes, while an additional 100 samples are reserved for testing, thus constituting the experimental dataset. The resolution stands at 321*321 pixels.

**Fig 2:**
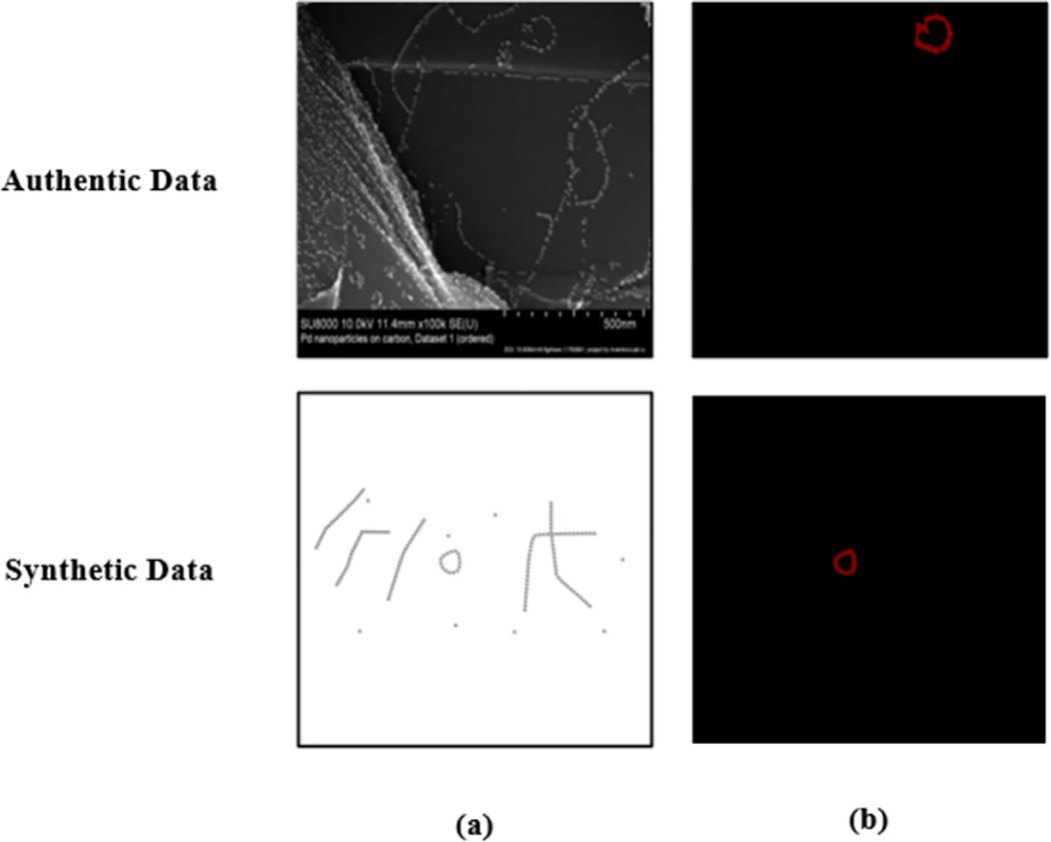
Illustrative comparison between AD and synthetic dataset labelled. (a) Original figure; (b) Marked images.

Within the training dataset, genuine and synthesized data are randomly sampled from pools comprising 510 and 850 instances, respectively, post data augmentation, in accordance with specific proportions. The selection of 100 authentic data points within the test set was conducted at random from a pool of 510 samples. Illustrative instances featuring real and synthetic data images alongside their corresponding labeled representations are depicted in Fig 2. Specifically, Fig 2(a) presents the unprocessed images of genuine and synthesized data, while Fig 2(b) showcases their labeled counterparts.

### Model selection

**Fig 3:**
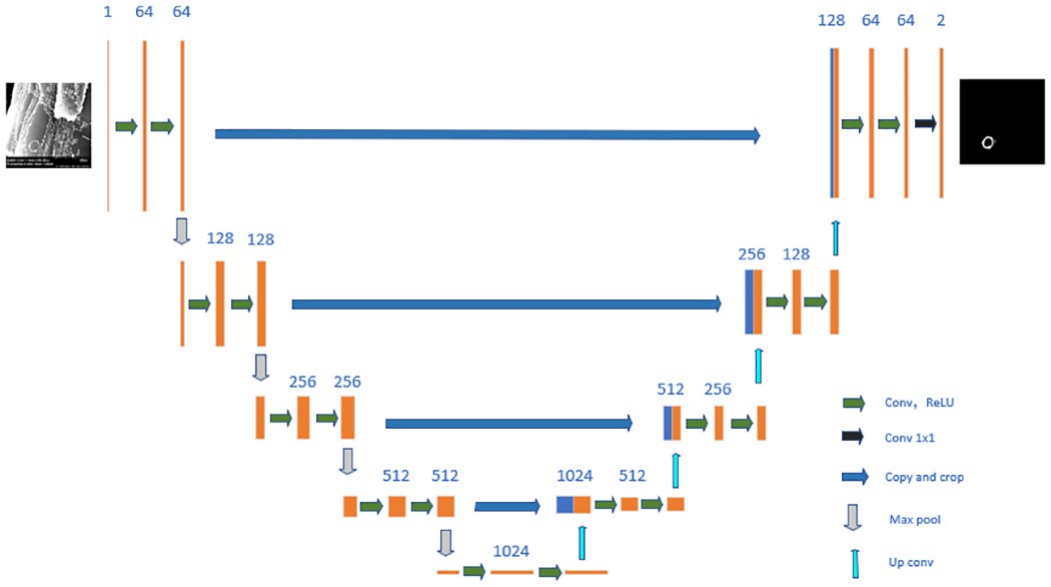
U-Net network diagram.

For the purpose of target segmentation and scene reconstruction, the detection of nanoparticles is imperative. We opted to employ the U-Net [39] model as the framework responsible for detecting the circular structure of each nanoparticle (e.g., see Fig 3), thereby facilitating a comparison with data enhancement algorithms. U-Net stands for a supervised learning approach widely employed in the realms of materials and medical image processing [11, 40, 41]. Operating as an encoder-decoder network, U-Net’s encoder segment conducts multiple convolution and pooling operations on the input image to extract and condense feature information, while the decoder segment primarily conducts up-sampling and deconvolution on the encoded feature information to gradually restore the image’s spatial resolution. A pivotal connection point exists at the midpoint between the encoder and decoder, known as the bottleneck layer, where information from the lower layers is extracted and shared as output, ensuring the preservation of crucial information. To prevent the loss of lower-level detail during encoding, U-Net employs skip connections, which also facilitate feature transfer from downsampling to upsampling, enabling direct transmission of bottom-layer information to the top layer and thus enhancing the network’s pixel localization accuracy. During the training phase, the models underwent co-training using batch gradient descent on a small batch comprising 10 images, with the ratio of real to SD not fixed. To ensure fairness, all models underwent training utilizing the same methodology, the details of which are outlined in the “Training setup” section.

### Evaluation indicators

Nanoparticle circular structures exemplify a segmentation task wherein a proficient algorithm must precisely detect and segment these circular structures in each image. Subsequent to segmentation, researchers extract and scrutinize the properties of the segmented nanoparticle circular structures to discern the correlation between microstructure and macroscopic material properties. In practical applications, various forms of noise may be introduced during sample preparation, significantly impacting the segmentation of nanoparticle circular structures. To effectively assess the algorithm’s performance, we employed several metrics, including Mean Intersection Over Union (MIoU) [42, 43], Kappa coefficient (Kappa) [44], Accuracy (Acc) [43], and Dice similarity index [45].

(1)MIoU [42, 43] (Mean Intersection Over Union) is a standard metric for semantic segmentation that calculates the average of the ratio of intersection to union across all categories. Below is its mathematical expression:

$$\begin{array}{c} \text{MioU}=\frac{1}{k+1}\sum_{i=0}^{k}\frac{p_{ii}}{\sum_{j=0}^{k}p_{ij}+\sum_{j=0}^{k}p_{ji}-p_{ii}} \end{array}$$ (1)

In this context, “i” represents the true value, “j” represents the predicted value, and “*p*_*ij*_” denotes the number of pixels that predict “i” to “j”. Thus, the numerator denotes the intersection between the true label and the predicted result, while the denominator signifies their union.

(2)The Kappa coefficient, as described by reference [44], serves as a metric for evaluating consistency and can additionally gauge the efficacy of classification. Consistency refers to the alignment between model predictions and actual classification outcomes. The Kappa coefficient ranges from -1 to 1, with 1 denoting perfect consistency, 0 indicating agreement consistent with a stochastic model, and -1 representing complete inconsistency. Generally, a Kappa coefficient falling between 0.4 and 0.6 signifies moderate agreement for most tasks, while values exceeding 0.6 indicate superior agreement. The formula for the Kappa coefficient is provided below:

$$\begin{array}{c} \text{Kappa}=\frac{p_{0}-p_{e}}{1-p_{e}} \end{array}$$ (2)

Here, “*p*_0_” denotes the observed proportion of perfect consistency, signifying the fraction of the total sample size where the model’s predictions precisely align with the actual observations. Conversely, “*p*_*e*_” represents the proportion of stochastic consistency, indicating the degree of agreement between the model’s predictions and actual observations that would be anticipated under complete randomness. In a dichotomous scenario, “*p*_*e*_” can be computed by summing the marginal probabilities of the two categories and multiplying them together.

(3)Acc referenced in [43], serves as a metric for assessing the efficacy of a classification model, representing the percentage of correct predictions made by the model out of the total number of predictions. Greater accuracy indicates a more proficient classifier. The formula for calculating accuracy is provided below:

$$\begin{array}{c} \text{Acc}=\frac{pred}{all}*100\% \end{array}$$ (3)

In this context, “pred” represents the count of samples correctly classified by the model, while “all” denotes the total number of sample predictions made by the model.

(4)The Dice similarity index, referenced in [45], functions as a measure of set similarity, employed to quantify the similarity between two samples. Frequently utilized for assessing the efficacy of segmentation algorithms, this index yields a score ranging from 1 (indicating optimal segmentation) to 0 (representing poor segmentation). Below is its mathematical expression:

$$\begin{array}{c} \text{Dice}=\frac{2*(pred\cap true)}{pred\cup true} \end{array}$$ (4)

In this context, “pred” refers to the set of predicted values, while “true” represents the set of true values. The numerator signifies the intersection between “pred” and “true,” which is then multiplied by 2 to account for the double counting of common elements between the two sets in the denominator. The denominator, on the other hand, represents the union of “pred” and “true.”

## Results and discussion

Throughout the training regimen, the data underwent random sampling. The training iterations spanned 100,000 cycles, with an initial learning rate established at 0.01. Stochastic Gradient Descent (SGD) served as the optimizer, while the DiceLoss function was adopted as the loss function, and the batch size was set to 16. The deep learning platform employed for this experiment was Baidu Flying Paddle, and detailed specifications of the hardware environment utilized are documented in Table 1. The construction, training, and testing of the network exclusively relied on the PaddleSeg framework [46] throughout the experiment. This framework provided comprehensive support, enhancing the efficiency and controllability of the experimental procedure.

**Table 1:** Environmental facilities.

Environmental	Version
Python	3.7.4
Paddlepaddle	2.2.2
GPU	3.7.4
Python	Tesla V100
Video Mem	32GB
CPU	4 Cores

To ascertain the ideal proportion of authentic to SD within the training dataset, initial experiments were performed exclusively on a dataset comprising solely AD, serving as a control condition for this investigation. Following this, trials were undertaken using a blend of AD and SD in varying ratios to ascertain the optimal fusion of the two. Subsequently, a suite of semantic segmentation models founded upon nanoparticle architectures underwent scrutiny, culminating in the selection of the U-Net model as the primary training framework. This iterative process was aimed at identifying the most efficacious data amalgamation for training, ensuring the proficient acquisition of knowledge pertaining to nanoparticle structures.

The findings from five experimental iterations conducted on a dataset comprising 400 AD are showcased in Table 2. The ensuing metrics, derived from averaging, encompass 0.8362 for Miou, 0.9967 for Acc, 0.8046 for Kappa, and 0.9023 for the Dice coefficient.

**Table 2:** 400 sheets of AD test results.

	Miou	Acc	Kappa	Dice
AD:400	0.8482	0.9969	0.8217	0.9108
AD:400	0.8304	0.9966	0.7966	0.8983
AD:400	0.8498	0.9970	0.8239	0.9119
AD:400	0.8204	0.9963	0.7820	0.8910
AD:400	0.8321	0.9967	0.7990	0.8995
Average	0.8362	0.9967	0.8046	0.9023

Table 3 illustrates the outcomes of experiments conducted on a proportional blend of AD and SD. Examination of the table reveals four experimental cohorts whose Miou metrics surpass the Miou of the control group, set at 0.8362. These four cohorts are as follows: 60 AD, 340 SD, with Miou recorded at 0.8517Acc at 0.9971, Kappa at 0.8264, and Dice coefficient at 0.9132; 85 AD, 315 SD, yielding Miou of 0.8391, Acc of 0.9967, Kappa of 0.8089, and Dice of 0.9045; 90 AD 310 SD, resulting in Miou of 0.8460, Acc of 0.9970, Kappa of 0.8186, and Dice of 0.9093; and finally, 100 AD, 300 SD, with Miou recorded at 0.8394, Acc at 0.9968, Kappa at 0.8093, and Dice coefficient at 0.9047.

**Table 3:** Test results for each scale of AD and SD.

	Miou	Acc	Kappa	Dice
AD:5;SD:395	0.7345	0.9945	0.6410	0.8205
AD:10;SD:390	0.7421	0.9949	0.6547	0.8273
AD:15;SD:385	0.7733	0.9955	0.7004	0.8542
AD:20;SD:380	0.7884	0.9957	0.7329	0.8665
AD:25;SD:375	0.8100	0.9963	0.7665	0.8832
AD:30;SD:370	0.8203	0.9964	0.7817	0.8909
AD:35;SD:365	0.8157	0.9964	0.7749	0.8875
AD:40;SD:360	0.8108	0.9964	0.7676	0.8838
AD:45;SD:355	0.8120	0.9963	0.7694	0.8847
AD:50;SD:340	0.8357	0.9968	0.8041	0.9021
AD:55;SD:345	0.8205	0.9966	0.7821	0.8911
AD:60;SD:340	0.8517	0.9971	0.8264	0.9132
AD:65;SD:335	0.8124	0.9963	0.7701	0.8850
AD:70;SD:330	0.8130	0.9964	0.7709	0.8854
AD:75;SD:325	0.8247	0.9966	0.7882	0.8941
AD:80;SD:320	0.8359	0.9967	0.8044	0.9022
AD:85;SD:315	0.8391	0.9967	0.8089	0.9045
AD:90;SD:310	0.8460	0.9970	0.8186	0.9093
AD:95;SD:305	0.8311	0.9965	0.7975	0.8988
AD:100;SD:300	0.8394	0.9968	0.8093	0.9047

Table 4 presents four experimental configurations that surpassed the control Miou. Five experiments were conducted for each of these configurations, and the resulting metrics were averaged. Notably, in the case of the experiment employing 60 instances of AD paired with 340 instances of SD, the Miou reached 0.8476, Acc attained 0.9970, Kappa achieved 0.8207, and Dice coefficient reached 0.9103. This particular experimental cohort exhibited the most superior performance, displaying a Miou increase of 0.0114 compared to the control group while utilizing the least amount of AD. Consequently, it was concluded that the optimal segmentation of the nanoparticle circular structure was achieved through the utilization of 60 instances of AD paired with 340 instances of SD.

**Table 4:** Results of five experiments for the four best results groups.

	Miou	Acc	Kappa	Dice
AD:60;SD:340	0.8319	0.9967	0.7986	0.8993
AD:60;SD:340	0.8517	0.9971	0.8264	0.9132
AD:60;SD:340	0.8533	0.9971	0.8286	0.9143
AD:60;SD:340	0.8528	0.9971	0.8279	0.9139
AD:60;SD:340	0.8484	0.9970	0.8219	0.9110
Average	0.8476	0.9970	0.8207	0.9103
AD:85;SD:315	0.8391	0.9967	0.8089	0.9045
AD:85;SD:315	0.8230	0.9965	0.7858	0.8929
AD:85;SD:315	0.8230	0.9965	0.7857	0.8929
AD:85;SD:315	0.8315	0.9967	0.7981	0.8990
AD:85;SD:315	0.8351	0.9966	0.8033	0.9016
Average	0.8303	0.9966	0.7964	0.8981
AD:90;SD:310	0.8460	0.9970	0.8186	0.9093
AD:90;SD:310	0.8380	0.9967	0.8074	0.9037
AD:90;SD:310	0.8397	0.9968	0.8098	0.9049
AD:90;SD:310	0.8262	0.9966	0.7905	0.8952
AD:90;SD:310	0.8314	0.9966	0.7980	0.8990
Average	0.8363	0.9967	0.8049	0.9024
AD:100;SD:300	0.8394	0.9968	0.8093	0.9047
AD:100;SD:300	0.8287	0.9965	0.7941	0.8970
AD:100;SD:300	0.8374	0.9968	0.8866	0.9033
AD:100;SD:300	0.8293	0.9966	0.7950	0.8975
AD:100;SD:300	0.8392	0.9968	0.8090	0.9045
Average	0.8348	0.9967	0.8188	0.9014

**Fig 4:**
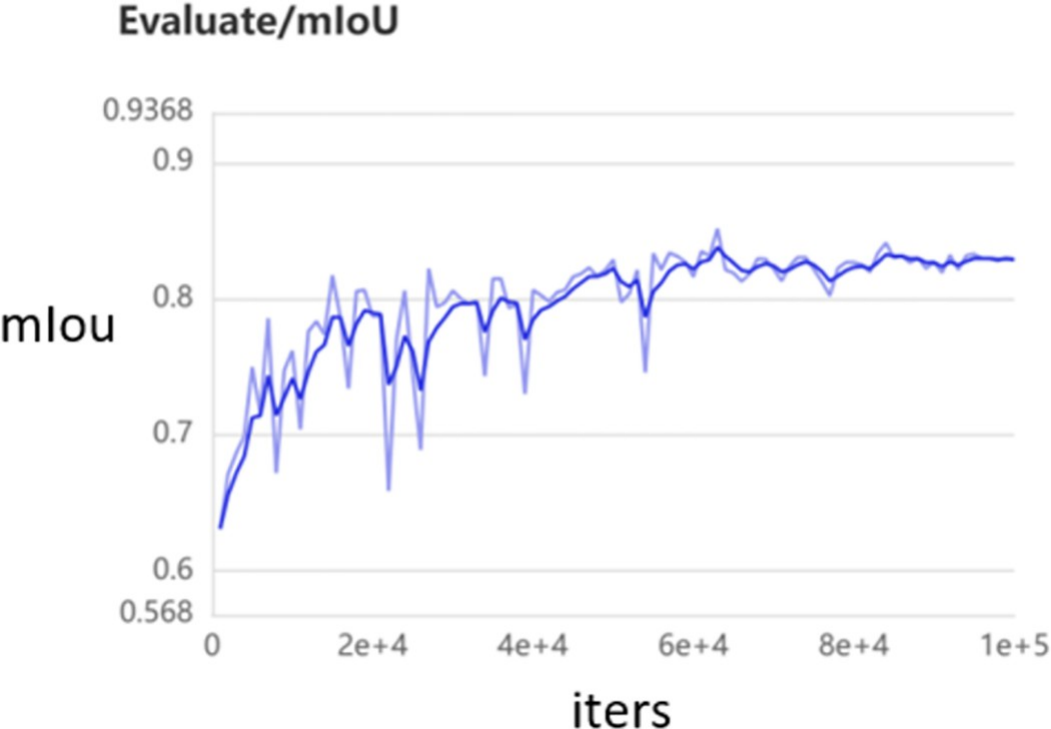
AD 60 sheets, SD 340 sheets assessed miou as 0.8533 fold plot.

Fig 4 depicts the line graph corresponding to the attainment of a Miou value of 0.8533 with 60 instances of AD and 340 instances of SD. Examination of the graph reveals the convergence of the Miou metric. The light blue line delineates the results recorded at intervals of 1000 iterations, while the dark blue line represents the smoothed curve derived from these results.

**Fig 5:**
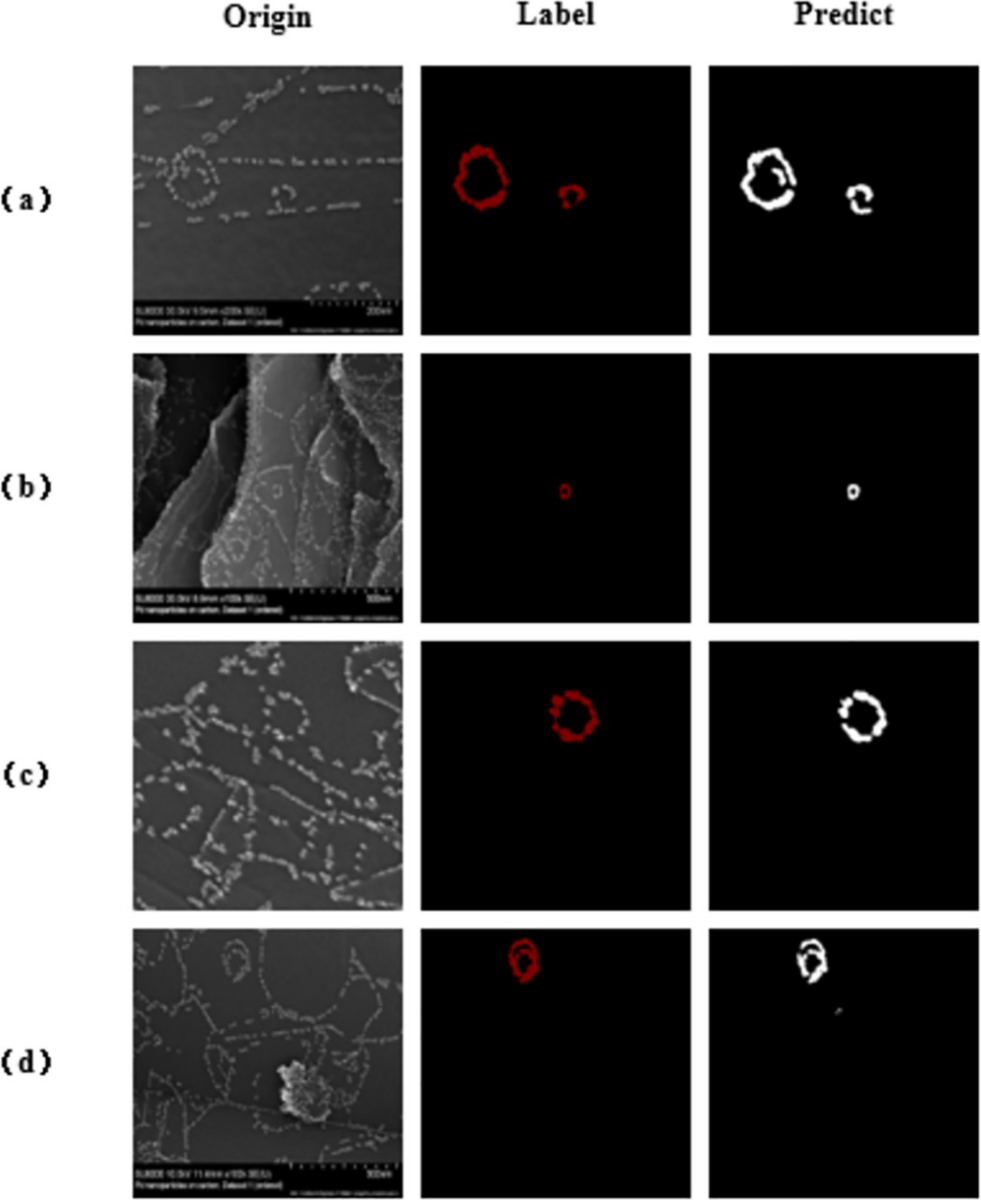
Prediction plot for AD 60 sheets, SD 340 sheets evaluating miou of 0.8533.

Fig 5 presents the prediction maps generated from an evaluation employing 60 AD instances and 340 SD instances, yielding a Miou score of 0.8533. Within the results, we have chosen four distinct images showcasing nanoparticle circular structures for analysis. Upon visual inspection, image a, characterized by a relatively uncomplicated background and simplistic line segments, exhibits a prediction that appears less optimal compared to image b. This discrepancy in prediction quality could stem from two potential factors: firstly, the possibility of nanoparticle adhesion, and secondly, despite the background simplicity, the presence of numerous scattered nanoparticles surrounding the central circle in image a. Conversely, image b portrays a background featuring intricate lines formed by nanoparticles, stark light-dark contrast, and prominent large particles. Despite the minor prominence of the nanoparticle circles within the overall composition, the predicted results exhibit minimal noise. Image c accentuates heightened nanoparticle adhesion compared to the surrounding area, evidenced by nearly every nanoparticle adhering to its neighbors. Meanwhile, image d presents a scenario where two circular structures overlap, with the larger circle enclosing the smaller one. Notably, our segmentation model adeptly distinguishes and delineates these structures. In summary, while image a’s background simplicity hints at potential factors influencing differences in prediction outcomes such as nanoparticle adhesion and fragmented background particles, images b and d underscore the model’s capacity to accurately predict and delineate nanoparticle structures even amidst complex backgrounds. Image c further highlights the prevalence of robust nanoparticle adhesion.

Table 5 presents ten semantic segmentation models (including Deeplabv3 [47], PspNet [48], CCNet [49], FastFCN [50], PFPNNet [51], GINet [52], ENCNet [53], BiseNet [54], etc.) after determining the ratio of real and SD, DaNet [55] and U2Net [56], etc.) were evaluated and the experimental results are shown in Table 5.

**Table 5:** Evaluation results of ten semantic segmentation models.

Models	Miou	Acc	Kappa	Dice
Deeplabv3	0.6395	0.9928	0.4419	0.7208
Pspnet	0.6983	0.9938	0.5712	0.7856
CCNet	0.7880	0.9956	0.7324	0.8662
FastFCN	0.7976	0.9956	0.7475	0.8737
PFPNNet	0.8405	0.9970	0.8109	0.9054
GINet	0.7383	0.9948	0.6478	0.8239
ENCNet	0.7859	0.9931	0.5868	0.7934
BiseNet	0.6104	0.9901	0.3699	0.6849
DaNet	0.6079	0.9924	0.3621	0.6807
U2Net	0.5847	0.9928	0.2976	0.6482

## Conclusion

In the realm of nanomaterials, researchers often rely on microscopy techniques to capture images of nanoparticle structures. However, this approach [8, 30, 32] presents several drawbacks, including its cumbersome nature, time-intensive process, and high associated costs. Particularly when dealing with a substantial volume of experimental data, the feasibility of employing microscopy techniques becomes significantly constrained. Hence, we propose a nanoparticle structure segmentation methodology grounded in SD. This approach entails constructing a dataset comprising a modest quantity of AD supplemented by a substantial volume of SD, thereby ensuring the requisite sample size and diversity essential for segmenting nanoparticle structures. By leveraging a limited quantity of authentic data, we can effectively assess the performance of the model or experimental system within realistic scenarios. Simultaneously, the integration of copious SD enhances dataset size and diversity, encompassing a broader array of scenarios and feature combinations, consequently rendering the results more universally applicable and robust. Among the 20 experimental iterations conducted, our findings indicate that employing 60 instances of authentic data and 340 instances of SD yields optimal results, surpassing those of the control group (comprising exclusively AD) across all assessment metrics. Lastly, we subjected an additional ten models to scrutiny for comparative analysis, ultimately selecting the U-Net model as the most suitable for nanoparticle structure segmentation, boasting Miou, Acc, Kappa, and Dice coefficients of 0.8476, 0.9970, 0.8207, and 0.9103, respectively.

The integration of authentic and synthetic datasets offers a potent and cost-efficient avenue for investigating nanoparticle structures, facilitating the attainment of desired experimental outcomes and the progression of scientific inquiry. Leveraging synthetic datasets for nanoparticle structure analysis circumvents numerous drawbacks, enhancing productivity, while deep learning methodologies exhibit superior adaptability, refining the techniques and processes employed in TEM nanoparticle image segmentation research.

## Supporting information

S1 Data Availability of data and material. (ZIP)

S1 Code Code used to build the model. (ZIP)
