# Supplementary material for: Segmentation study of nanoparticle topological structures based on synthetic data
Source: PLoS One. 2024 Oct 2;19(10):e0311228. doi: 10.1371/journal.pone.0311228 (PMC11446430; doi:10.1371/journal.pone.0311228)
Supplement: S1 Code — (ZIP) [file pone.0311228.s002.zip › S1 Code/main.docx]

The deep learning work in this paper was completed in Baidu's PaddlePaddle, with the construction, training, and testing of the network entirely based on the PaddleSeg framework.In this context, the data needs to be placed in the 'work' directory.

Main:

# unzip

!unzip -oq /home/aistudio/data/data280245/PaddleSeg.zip -d work

# train

!export CUDA_VISIBLE_DEVICES=0

!python PaddleSeg/train.py \

       --config unet.yml \

       --do_eval \

       --use_vdl \

       --save_interval 1000 \

       --save_dir output

# validate

!python PaddleSeg/val.py \

       --config unet.yml \

       --model_path output/best_model/model.pdparams
