# Supplementary material for: Segmentation study of nanoparticle topological structures based on synthetic data
Source: PLoS One. 2024 Oct 2;19(10):e0311228. doi: 10.1371/journal.pone.0311228 (PMC11446430; doi:10.1371/journal.pone.0311228)
Supplement: S1 Code — (ZIP) [file pone.0311228.s002.zip › S1 Code/PaddleSeg.zip!/PaddleSeg/deploy/web/example/index.html]

paddle web demo - humanseg


loading...
