# Supplementary material for: Segmentation study of nanoparticle topological structures based on synthetic data
Source: PLoS One. 2024 Oct 2;19(10):e0311228. doi: 10.1371/journal.pone.0311228 (PMC11446430; doi:10.1371/journal.pone.0311228)
Supplement: S1 Code — (ZIP) [file pone.0311228.s002.zip › S1 Code/PaddleSeg.zip!/PaddleSeg/EISeg/README_AR.md]

# EISeg
[![Python 3.6](https://camo.githubusercontent.com/75b8738e1bdfe8a832711925abbc3bd449c1e7e9260c870153ec761cad8dde40/68747470733a2f2f696d672e736869656c64732e696f2f62616467652f707974686f6e2d332e362b2d626c75652e737667)](https://www.python.org/downloads/release/python-360/) [![PaddlePaddle 2.2](https://camo.githubusercontent.com/f792707056617d58db17dca769c9a62832156e183b6eb29dde812b34123c2b18/68747470733a2f2f696d672e736869656c64732e696f2f62616467652f706164646c65706164646c652d322e322d626c75652e737667)](https://www.python.org/downloads/release/python-360/) [![License](https://camo.githubusercontent.com/9330efc6e55b251db7966bffaec1bd48e3aae79348121f596d541991cfec8858/68747470733a2f2f696d672e736869656c64732e696f2f62616467652f6c6963656e73652d417061636865253230322d626c75652e737667)](https://github.com/PaddlePaddle/PaddleSeg/blob/develop/EISeg/LICENSE) [![Downloads](https://camo.githubusercontent.com/d3d7e08bac205f34cee998959f85ffcbe6a9aca4129c20d7a5ec449848826d48/68747470733a2f2f706570792e746563682f62616467652f6569736567)](https://pepy.tech/project/eiseg)
[Chinese (Simplified)](README.md) | [English](README\_EN.md) | عربي
## آخر التطورات
- ورقتنا البحثية عن ال (interactive segmentation) بأسم [EdgeFlow](https://arxiv.org/abs/2109.09406) تم قبولها من قبل ICCV 2021 Workshop.
- دعم الاستدلال على الرسم البياني الثابت مع سرعة تفاعل واداء عالي بالكامل ؛ إطلاق أحدث إصدار من EISeg 0.4.0 والذي يضيف حديثًا الاستشعار عن بعد ، ووضع العلامات الطبية ، وتقسيم الصور كبيرة الحجم الى اجزاء صغيره ليمكن استخدامها.
## مقدمة
EISeg (Efficient Interactive Segmentation), تم بنائه على أساس [RITM](https://github.com/saic-vul/ritm\_interactive\_segmentation) و [EdgeFlow](https://arxiv.org/abs/2109.09406), هو برنامج تعليمي فعال وذكي للتجزئة تم تطويره على أساس PaddlePaddle. إنه يغطي عددًا كبيرًا من نماذج التجزئة عالية الجودة في اتجاهات مختلفة مثل الصور بانواعها العامة ، والصورة الشخصية ، والاستشعار عن بعد ، والعلاج الطبي ، وما إلى ذلك ، مما يوفر الراحة للتعليق التوضيحي السريع للملصقات الدلالية والمثيلات بتكلفة منخفضة. بالإضافة إلى ذلك ، من خلال تطبيق التعليقات التوضيحية التي حصل عليها EISeg على نماذج التجزئة الأخرى المقدمة من PaddleSeg للتدريب ، يمكن إنشاء نماذج عالية الأداء مع حالات مخصصة ، ودمج العملية الكاملة لمهام التجزئة من شرح البيانات إلى تدريب النموذج والاستدلال.
[![4a9ed-a91y1](https://user-images.githubusercontent.com/71769312/141130688-e1529c27-aba8-4bf7-aad8-dda49808c5c7.gif)](https://user-images.githubusercontent.com/71769312/141130688-e1529c27-aba8-4bf7-aad8-dda49808c5c7.gif)
## تجهيز النموذج
يرجى تنزيل معلمات النموذج قبل استخدام EIseg. يوفر EISeg 0.4.0 أربعة نماذج اتجاه مدربة على COCO + LVIS ، وبيانات صورة واسعة النطاق ، و mapping\_challenge ، و LiTS (تحدي تجزئة ورم الكبد) لتلبية احتياجات وضع العلامات للسيناريوهات العامة والصورة بالإضافة إلى الهندسة المعمارية والكبد في الصور الطبية. تتوافق بنية النموذج مع وحدة اختيار الشبكة في أدوات EISeg التفاعلية ، ويحتاج المستخدمون إلى تحديد هياكل شبكة مختلفة ومعلمات التحميل وفقًا لاحتياجاتهم الخاصة.
| نوع النموذج | سيناريوهات الاستخدام | معمارية النموذج | رابط التنزيل |
| ---------------------- | ---------------------------------------- | ------------------ | ------------------------------------------------------------ |
| High Performance Model | تحديد الصور العامة | HRNet18\_OCR64 | [static\_hrnet18\_ocr64\_cocolvis](https://paddleseg.bj.bcebos.com/eiseg/0.4/static\_hrnet18\_ocr64\_cocolvis.zip) |
| Lightweight Model | تحديد الصور العامة | HRNet18s\_OCR48 | [static\_hrnet18s\_ocr48\_cocolvis](https://paddleseg.bj.bcebos.com/eiseg/0.4/static\_hrnet18s\_ocr48\_cocolvis.zip) |
| High Performance Model | تحديد الصور في وضع ال portrait | HRNet18\_OCR64 | [static\_hrnet18\_ocr64\_human](https://paddleseg.bj.bcebos.com/eiseg/0.4/static\_hrnet18\_ocr64\_human.zip) |
| Lightweight Model | تحديد الصور في وضع ال portrait | HRNet18s\_OCR48 | [static\_hrnet18s\_ocr48\_human](https://paddleseg.bj.bcebos.com/eiseg/0.4/static\_hrnet18s\_ocr48\_human.zip) |
| High Performance Model | تحديد الصور العامة | EdgeFlow | [static\_edgeflow\_cocolvis](https://paddleseg.bj.bcebos.com/eiseg/0.4/static\_edgeflow\_cocolvis.zip) |
| Lightweight Model | تحديد صور الاستشعار عن بعد | HRNet18s\_OCR48 | [static\_hrnet18\_ocr48\_rsbuilding\_instance](https://paddleseg.bj.bcebos.com/eiseg/0.4/static\_hrnet18\_ocr48\_rsbuilding\_instance.zip) |
| Lightweight Model | تحديد الصور الطبية والكبد | HRNet18s\_OCR48 | [static\_hrnet18s\_ocr48\_lits](https://paddleseg.bj.bcebos.com/eiseg/0.4/static\_hrnet18s\_ocr48\_lits.zip) |
\*\*ملاحظة\*\* : يجب وضع بنية النموذج الذي تم تنزيله "\* .pdmodel" ومعلمات النموذج المقابلة "\* .pdiparams" في نفس مسار المجلد على جهازك. عند تحميل النموذج ، ما عليك سوى تحديد موقع معلمة النموذج في نهاية `\* .pdiparams` ، وسيتم تحميل` \* .pdmodel` تلقائيًا. عند استخدام نموذج "EdgeFlow" ، يرجى إيقاف تشغيل "استخدام قناع" والتحقق من "استخدام قناع" عند استخدام طرز أخرى.
## كيفية التثبيت \ التنصيب
يوفر EISeg طرقًا متعددة للتثبيت ، من بينها [pip](#PIP) و [كود التشغيل](#كودالتشغيل) متوافقان مع أنظمة التشغيل Windows و Mac OS و Linux. يوصى بالتثبيت في بيئة افتراضية تم إنشاؤها بواسطة Conda خوفًا من مشاكل في عدم التوافق.
متطلبات الإصدار:
- PaddlePaddle >= 2.2.0
لمزيد من التفاصيل حول تثبيت PaddlePaddle ، يرجى الرجوع إلى موقعنا [الموقع الرسمي](https://www.paddlepaddle.org.cn/install/quick?docurl=/documentation/docs/zh/install/pip/windows-pip.html).
### Clone
استنساخ PaddleSeg الي جهازك عن طريق git:
```
git clone https://github.com/PaddlePaddle/PaddleSeg.git
```
قم بتثبيت البيئة المطلوبة (إذا كنت بحاجة إلى استخدام GDAL و SimpleITK ، فيرجى الرجوع إلى \*\*Vertical Segmentation\*\* للتثبيت).
```
pip install -r requirements.txt
```
قم بتمكين EISeg عن طريق تشغيل eiseg بعد تثبيت البيئة المطلوبة:
```
cd PaddleSeg\EISeg
python -m eiseg
```
أو يمكنك تشغيل exe.py في eiseg:
```
cd PaddleSeg\EISeg\eiseg
python exe.py
```
### PIP
قم بتثبيت pip على النحو التالي ：
```
pip install eiseg
```
تقوم pip بتثبيت التبعيات تلقائيًا. بعد ذلك ، أدخل ما يلي في سطر command line:
```
eiseg
```
الآن ، يمكنك تشغيل pip.
## الأستخدام
بعد فتح البرنامج ، قم بإجراء الإعدادات التالية قبل التحديد على الصور:
1. \*\*تحميل نموذج المعلمة\*\*
حدد الشبكة المناسبة وقم بتحميل معلمات النموذج المقابلة. يشهد EISeg0.4.0 تحويل استدلال الرسم البياني الديناميكي إلى استنتاج ثابت وتحسينات شاملة لسرعة الاستدلال بنقرة واحدة. بعد تنزيل وفك ضغط النموذج والمعلمات الصحيحة ، يجب وضع بنية النموذج `\* .pdmodel` ومعلمات النموذج المقابلة` \* .pdiparams` في نفس الدليل ، وفقط موقع معلمة النموذج في نهاية `\* يجب تحديد .pdiparams` عند تحميل النموذج. تستغرق تهيئة النموذج الثابت بعض الوقت ، يرجى الانتظار بصبر حتى يتم تحميل النموذج. سيتم تسجيل معلمات النموذج التي تم تحميلها بشكل صحيح في `معلمات النموذج الحديثة` ، والتي يمكن تبديلها بسهولة ، وسيتم تحميل معلمة النموذج الخارج تلقائيًا في المرة التالية التي تفتح فيها البرنامج.
2. \*\*فتح الصور\*\*
قم بفتح مجلد الصور التي تحتوي الصور المراد تحديدها وعند تحميلها نلاحظ ظهورها في الشاشة الرئيسية للبرنامج تحت `قائمة البيانات`.
3. \*\*اضافة او إستيراد علامات\*\*
إضافة / تحميل العلامات. يمكن إنشاء علامات جديدة عن طريق `إضافة تسمية` ، والتي تنقسم إلى 4 أعمدة مطابقة لقيمة البكسل والوصف واللون والحذف. يمكن حفظ العلامات التي تم إنشاؤها حديثًا كملفات txt عن طريق `حفظ قائمة العلامات` ، ويمكن للمتعاونين الآخرين استيراد العلامات عن طريق `تحميل قائمة العلامات`. العلامات التي تم استيرادها عن طريق التحميل سيتم تحميلها تلقائيًا بعد إعادة تشغيل البرنامج.
4. \*\*الحفظ التلقائي\*\*
يمكنك اختيار مجلد الحفظ المناسب وتشغيل `الحفظ التلقائي`, بحيث يتم حفظ الصورة التي انتهيت منها تلقائيًا عند تبديل الصور.
يمكنك البدأ في استخدام البرنامج الان عندما تتم أعداد كل ما سبق, يوجد ايضًا اختصارات للوحة المفاتيح لتسهل عليك ويمكن تعديلها عن طريق الضغط على حرف `E` بم يناسبك.
| المفتاح الو الحروف الدالة على الاختصارات | الخاصية |
| --------------------------------- | ------------------------------ |
| زر الفأرة الايسر | أضف نقاط عينة موجبة |
| زر الماوس الايمن | أضف نقاط عينة سلبية |
| زر الماوس الأوسط | تحريك الصورة |
| Ctrl + زر الماوس الأوسط （عجلة) | تكبير الصورة |
| S | الصورة السابقة |
| F | الصورة التالية |
| مسافة | انهاء التعليم |
| Ctrl+Z | خطوة للخلف |
| Ctrl+Shift+Z | مسح |
| Ctrl+Y | خطوة للأمام |
| Ctrl+A | فتح صورة |
| Shift+A | افتح المجلد |
| E | افتح قائمة مفاتيح الاختصار |
| Backspace | حذف المضلع |
| نقرتين متتاليتين（نقطة）| حذف نقطة |
| نقرتين متتاليتين（حرف）| إضافة نقطة |
## تعليمات الخاصيات الجديدة
- \*\*مضلع\*\*
- انقر فوق مفتاح ال `مسافة` لإكمال التعليق التوضيحي التفاعلي ، ثم يظهر حدود المضلع.
- عندما تحتاج إلى متابعة العملية التفاعلية داخل المضلع ، انقر فوق `مسافة` للتبديل إلى الوضع التفاعلي حتى لا يمكن تحديد المضلع وتغييره.
- يمكن حذف المضلع. استخدم الماوس الأيسر لسحب نقطة الربط ، وانقر نقرًا مزدوجًا فوق نقطة الربط لحذفها ، ثم انقر نقرًا مزدوجًا فوق أحد الجوانب لإضافة نقطة ربط.
- مع تشغيل `الاحتفاظ بأقصى عدد من الكتل المتصلة` ، ستبقى المساحة الأكبر فقط في الصورة ، ولن يتم عرض باقي المساحات الصغيرة وحفظها.
- \*\*الصيغات المختلفة للحفظ واخراج الملفات\*\*
- سيتم تسجيل المضلعات وتحميلها تلقائيًا بعد إعداد `حفظ JSON` أو `COCO حفظ`.
- مع عدم وجود مسار حفظ محدد ، يتم حفظ الصورة في مجلد التسمية ضمن مجلد الصورة الحالي افتراضيًا.
- إذا كانت هناك صور بنفس الاسم ولكن بصيغة مختلفة ، فيمكنك فتح `التسميات والصور ذات الامتدادات نفسها`.
- يمكنك أيضًا الحفظ كصورة ذات تدرج رمادي أو ألوان زائفة أو غير لامعة ، راجع الأدوات 7-9 في شريط الأدوات.
- \*\*استخدام واخراج القناع\*\*
- يمكن سحب الملصقات عن طريق الضغط باستمرار على العمود الثاني ، وسيتم الكتابة فوق القناع النهائي الذي تم إنشاؤه من أعلى إلى أسفل وفقًا لقائمة العلامات.
- \*\*وحدة واجهة\*\*
- يمكنك تحديد وحدة الواجهة ليتم عرضها في `العرض` ، وسيتم تسجيل حالة الخروج العادية وموقع وحدة الواجهة ، وتحميلها تلقائيًا عند فتحها في المرة القادمة.
- \*\*تجزئة رأسية\*\*
يدعم EISeg الآن صور الاستشعار عن بعد وتجزئة الصور الطبية ، ويلزم تثبيت تبعيات إضافية لتشغيلها.
- قم بتثبيت GDAL للاستشعار عن بعد لتجزئة الصورة ، يرجى الرجوع إلى [تجزئة الاستشعار عن بعد](docs/remote\_sensing\_en.md)。
- قم بتثبيت SimpleITK لتجزئة الصور الطبية ، يرجى الرجوع إلى [تجزئة الصور الطبية](docs/medical\_en.md)。
- \*\*أداة البرمجة النصية\*\*
يوفر EISeg حاليًا أدوات البرمجة النصية بما في ذلك التعليقات التوضيحية على مجموعة بيانات PaddleX ، وتحديد تنسيق COCO والتسميات الدلالية لتسميات المثيلات ، إلخ. راجع [استخدام أدوات البرمجة النصية](https://github.com/PaddlePaddle/PaddleSeg/blob/develop/EISeg/) لمزيد من التفاصيل.
## تحديثات الإصدار
يمكنك نعرفة آخر التحديقات من [هنا](README\_EN.md#version-updates)
## المساهمون
نشكر المطورين [Yuying Hao](https://github.com/haoyuying), [Lin Han](https://github.com/linhandev), [Yizhou Chen](https://github.com/geoyee), [Yiakwy](https://github.com/yiakwy), [GT](https://github.com/GT-ZhangAcer), [Youssef Harby](https://github.com/Youssef-Harby), [Zhiliang Yu](https://github.com/yzl19940819), [Nick Nie](https://github.com/niecongchong) والدعم من قبل [RITM](https://github.com/saic-vul/ritm\_interactive\_segmentation).
## الاقتباس
إذا وجدت مشروعنا مفيدًا في بحثك ، فيرجى كتابة الاقتباس :
```
@article{hao2021edgeflow,
title={EdgeFlow: Achieving Practical Interactive Segmentation with Edge-Guided Flow},
author={Hao, Yuying and Liu, Yi and Wu, Zewu and Han, Lin and Chen, Yizhou and Chen, Guowei and Chu, Lutao and Tang, Shiyu and Yu, Zhiliang and Chen, Zeyu and others},
journal={arXiv preprint arXiv:2109.09406},
year={2021}
}
```
